# Broadband, Wide‐Angle, and Versatile Metasurface Illusions with Inverse Synthetic Aperture Radar Imaging

**DOI:** 10.1002/advs.202416172

**Published:** 2025-02-08

**Authors:** Haoran Li, Kang Luo, Din Ping Tsai, Yulang Li, Linyuan Dou, Shuqiao Li, Tao He, Yuancheng Fan, Mu Ku Chen, Zhanshan Wang, Yuzhi Shi, Zeyong Wei, Xinbin Cheng

**Affiliations:** ^1^ Institute of Precision Optical Engineering School of Physics Science and Engineering Tongji University Shanghai 200092 China; ^2^ MOE Key Laboratory of Advanced Micro‐Structured Materials Shanghai 200092 China; ^3^ Shanghai Institute of Intelligent Science and Technology Tongji University Shanghai 200092 China; ^4^ Shanghai Frontiers Science Center of Digital Optics Shanghai 200092 China; ^5^ National Key Laboratory of Electromagnetic Energy Naval University of Engineering Wuhan 430033 China; ^6^ Department of Electrical Engineering City University of Hong Kong Kowloon Hong Kong 999077 China; ^7^ State Key Laboratory of Terahertz and Millimeter Waves City University of Hong Kong Kowloon Hong Kong 999077 China; ^8^ Centre for Biosystems, Neuroscience, and Nanotechnology City University of Hong Kong Kowloon Hong Kong 999077 China; ^9^ MOE Key Laboratory of Material Physics and Chemistry under Extraordinary Conditions School of Physical Science and Technology Northwestern Polytechnical University Xi'an 710129 China

**Keywords:** illusions, metasurfaces, microwaves, radar

## Abstract

Generation of controllable illusions has raised widespread interest. Over the past few decades, this field has been revolutionized by the emergence of metamaterials and metasurfaces. However, current efforts utilizing single‐layer metasurfaces are limited to simple illusion demonstrations by reproducing electromagnetic field distributions, which also struggle to achieve both broad bandwidths and wide angular ranges. Here, a nontrivial multi‐layer illusion strategy is proposed utilizing the inverse synthetic aperture radar imaging. The customization of illusions is facilitated by correlating the phase dispersion of meta‐atoms within the target frequency interval with the positioning of the structure. The approach enables the creation of illusions across a broadband of 8 to 12 GHz and a wide angular range from −30° to 30°. As a proof of principle, several illusions are experimentally demonstrated, showcasing the diversity of the approach. This work presents a viable illusion strategy that is more feasible for practical applications in the microwave domain, paving the way for future advances of customizable illusions.

## Graphical abstract

“Illusion” is a captivating topic throughout human civilization. This research proposes an advanced multi‐layer metasurface illusion strategy utilizing a technique known as “inverse synthetic aperture radar (ISAR)” imaging, enabling the creation of illusions over broadband and wide‐angle. This work demonstrates powerful metasurface illusion functions, such as misalignment, splitting, bending, and tilting of a metal flat plate.

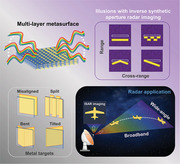

## 1 Introduction

“Illusion” is a captivating topic throughout human civilization. It refers to a camouflage technique that allows the human eyes or electromagnetic wave detectors to perceive one object as another, perfectly illustrating the concept of “what you see is not what you get”. The capability to make objects exhibit envisioned illusions has been a long‐standing dream of humanity. The academic study of illusion was first theoretically proposed in 2009, involving the cancellation of the original object using a “complementary medium” and restoring a different object through a “restoring medium”.^[^ ^1^ ^]^ However, the anisotropic and inhomogeneous nature of extreme materials used in the study makes it difficult to realize in reality.^[^ ^2^, ^3^ ^]^ In addition, bulky weights, complex manufacturing processes, and high cost hindered their applications.

Metasurfaces are a 2D form of metamaterials with advantages including ultrathin thickness, light‐weight, and powerful capabilities in steering electromagnetic waves. The phase, amplitude, and polarization of the electromagnetic field can be manipulated with the metasurface, demonstrating its strong capabilities in a great diversity of fascinating phenomena such as anomalous refraction and reflection,^[^ ^4^, ^5^, ^6^, ^7^ ^]^ invisibility cloak,^[^ ^8^, ^9^, ^10^ ^]^ metalenses,^[^ ^11^, ^12^, ^13^ ^]^ holograms,^[^ ^14^, ^15^, ^16^, ^17^ ^]^ optical manipulation,^[^ ^18^, ^19^, ^20^ ^]^ and many others.^[^ ^21^, ^22^, ^23^, ^24^, ^25^, ^26^ ^]^ Invisibility is generally regarded as a special form of illusion, where the scattered field influenced by the target is remodulated into free space through the design of an invisibility cloak.^[^ ^27^, ^28^, ^29^ ^]^ In recent years, theories and researches on invisibility cloaks have advanced significantly, leading to numerous applications tailored to various scenarios.^[^ ^30^, ^31^, ^32^, ^33^, ^34^, ^35^ ^]^ In the last several years, advances in metasurfaces have rekindled the enthusiasm for illusion researches with tremendous progress.^[^ ^36^, ^37^, ^38^, ^39^, ^40^, ^41^, ^42^ ^]^ By covering a hidden region or object with a deliberately designed metasurface, it is possible to reconstruct the scattering field of illusion. However, conventional methods for manipulating electromagnetic field distributions in both the near or far field are difficult to achieve various designated illusions. This makes the existing illusion studies face significant challenges in the illusion design and practical applications.

In addition, most reported illusion single‐layer metasurfaces typically operate only within narrow frequency bands and at specific incidence angles, as they are pre‐designed for a target frequency with a defined phase gradient. When the frequency or incident angle deviates from the design, performances of the illusions cannot be guaranteed due to the inherent dispersions of resonant meta‐atoms. Though the broadband metasurface illusion has been reported, it works at normal incidence and demonstrates only the focal point array.^[^ ^43^ ^]^ Developing broadband, wide‐angle, and versatile metasurface illusions is crucial in various practical applications, yet it remains a formidable challenge.

Here, we propose a nontrivial illusion concept based on the inverse synthetic aperture radar (ISAR) imaging process, which is employed in radar applications.^[^ ^44^, ^45^ ^]^ By processing the scattering information over a broadband and wide‐angle range, a 2D ISAR image containing important feature information such as the shape and position of the target can be obtained, enabling direct target identification. The relationship between the broadband phase dispersion of meta‐atoms and the position of the illusions in ISAR image is constructed. To achieve the desired phase dispersion over broadband and high reflection at normal incidence, resonant meta‐atoms in three layers are designed, through which the illusion in ISAR image can be precisely and freely controlled. Illusions are reconstructed and determined by collecting the broadband wide‐angle scattering fields. Various illusion examples of misalignment, splitting, bending, and tilting of metal flat plates are demonstrated by metasurfaces in broadband (8 to 12 GHz) and wide‐angle (−30° to 30°) ISAR imaging. Our work bridges existing illusion strategies with practical application scenarios, making significant progress in illusion design for broadband and wide‐angle performance from an imaging perspective.

## 2 Results and Discussion

### 2.1 Physical Mechanism of the Metasurface Illusion with ISAR Imaging

**Figure 1:**
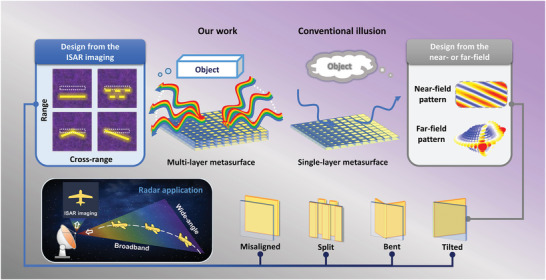
Schematic of a customized illusion achieved by designing a multi‐layer metasurface based on ISAR imaging. ISAR imaging is one of the common radar applications that collects the scattered field of a target for imaging in a wide bandwidth and at a wide angular range, allowing direct observation of the target's structural features. Incident waves with a wide bandwidth and wide angular range are illuminated onto the designed metasurface. Using a multi‐layer metasurface, ISAR imaging of the scattered data allows desired design illusions to be observed in the image, such as the misaligned, split, bent, and tilted plates. Conventional illusions typically use a single‐layer metasurface to reproduce the scattering pattern in the near and far fields of the target, resulting in a very limited illusionary effect.

The schematic diagram of the illusion metasurface is shown in **Figure** **1**. Conventional illusion metasurface (right panel of Figure 1) are designed for fixed frequencies and thus can only exhibit illusionary effects at the designed frequency. An ordered arrangement of the meta‐atoms is used to modulate the scattering field and reproduce the wavefront distortion produced by the target. The quality of the illusion is evaluated by scattering patterns in near and far fields. Due to the inherent phase dispersion limitations of the material, the illusion effect is significantly diminished when the conventional metasurfaces are under the illumination of a wide‐bandwidth electromagnetic waves.

Radar imaging is a vital tool for enhancing human perception and deepening our understanding of the physical world.^[^ ^46^ ^]^ It is less affected by weather and lighting conditions than optical imaging. ISAR is a widely used radar imaging technique that generates images by coherently processing the received radar echoes from transmitted pulses. Unlike photographic images that contain only the amplitude information of the target's light reflectivity, ISAR delivers both the amplitude and the phase information of the target's electromagnetic scattering field.^[^ ^47^ ^]^ The phase and amplitude distributions of the broadband and wide‐angle range scattered fields are used to realize range and cross‐range profiles in ISAR imaging, respectively. The accuracy of the observed target is closely related to the available spatial resolution, while the spatial resolution of ISAR image using the Fourier transform depends mainly on the frequency bandwidth and the integration angle domain.^[^ ^48^ ^]^ High resolution in the range direction is achieved by transmitting pulses within a large bandwidth, while the high resolution in the cross‐range direction is achieved by utilizing the relative motion between the radar and the target to form a synthetic aperture.^[^ ^49^, ^50^ ^]^

The illusion metasurface based on ISAR imaging is shown on the left side of Figure 1. Multi‐layer meta‐atoms with multi‐resonances are employed for the metasurface to achieve an orderly adjustment of the scattered field distribution in broadband and wide‐angle. ISAR images for the illusion are obtained by collecting broadband and wide‐angle scattered field data, performing inverse Fourier transform, and post‐processing the collected data. The method allows for designing various illusion modes, such as misalignment, splitting, bending, and tilting of flat metal plates, which are more powerful than conventional illusions that typically only showcase the result of plate tilting.

In broadband applications, the desired phase distribution depends not only on the spatial position but also on the operation frequency. To achieve the desired illusion across a broad bandwidth, meta‐atoms must provide a large phase range, favorable phase dispersion within the operating frequency range, and high amplitude under normal incidence. In principle, the higher‐order phase response of meta‐atoms is crucial for realizing broadband applications.^[^ ^13^ ^]^ However, single‐layer meta‐atoms have limited ability to regulate phase dispersion due to their resonant properties, thus hindering the realization of broadband illusions. Distinctively, meta‐atoms composed of multiple metallic layers with material spacer layers can extend the variation range of the reflection phase transition through mutual interactions among multi‐resonant systems, which has been commonly used in broadband studies.^[^ ^51^, ^52^, ^53^, ^54^, ^55^, ^56^ ^]^

### 2.2 Design Process of the Metasurface Illusion

**Figure 2:**
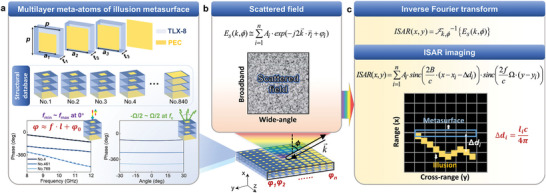
Schematic diagram of the metasurface and physical mechanisms used to realize the illusion with ISAR imaging. a) Schematic of meta‐atoms and four metallic layers separated by three TLX‐8 spacers (*ε_r_* = 2.55 + 0.0017*i*). *p* = 8 mm, *t_1_* = *t_2_* = *t_3_* = 1.6 mm; *a_1_*, *a_2_*, *a_3_* are appropriately tuned for each designing unit cell. The phase distributions of the meta‐atoms at different frequencies are obtained at an incidence angle of 0°. The phase distributions of the meta‐atoms at different angles are obtained at the center frequency. The direction of observation is the same as the direction of incidence. b) Modulation of the scattered field from the illusion metasurface. Meta‐atoms at different locations have different phase distribution characteristics. c) Computational process for realizing the metasurface illusion and schematic diagram of illusion in ISAR imaging.

Considering required phase properties of the reflective metasurface and the complexity of the final structure, we choose a multi‐resonant meta‐atom consisting of four perfect‐electronic‐conductor (PEC) layers separated by three TLX‐8 material spacer layers (*ε_r_* = 2.55 + 0.0017*i*) for the reflective metasurface, as shown in **Figure** **2**a. The period of the meta‐atom is *p* = 8 mm, the thickness of each TLX‐8 material spacer is *t_1_* = *t_2_* = *t_3_* = 1.6 mm, and the thickness of each metallic layer is 0.018 mm. By varying structure parameters *a_1_*, *a_2_*, and *a_3_* for each unit cell (ranging from 1 to 7.9 mm with a variation interval of 0.1 mm, keeping *a_1_* ≤ *a_2_* ≤ *a_3_*), we create a database consisting of 840 different structures, and numbered them in the database. For example, for No. 4, *a_1_* = 1 mm, *a_2_* = 1 mm and *a_3_* = 1.3 mm; for No. 461, *a_1_* = 2 mm, *a_2_* = 6 mm and *a_3_* = 6 mm; for No. 769, *a_1_* = 5 mm, *a_2_* = 5 mm and *a_3_* = 7.8 mm. Different meta‐atoms (No. 4, No. 461, No. 769) show relatively stable linear phase distributions over a wide bandwidth from 8 to 12 GHz, which can be expressed by *φ* ≈ *f* · *l* + *φ_0_* (where *φ* ≈ *f* · *l* represents the portion of the phase that transforms linearly with frequency *f* and *l* represents the linear parameter; *φ_0_* represents the portion of the phase that is independent of frequency change). Moreover, the phase of the meta‐atom exhibits a slow variation with the angle of incidence within the range of 0°–30°. For such a resonant meta‐atom, the phase response is insensitive to angular changes.^[^ ^10^, ^54^ ^]^ This makes the illusion strategy effective at oblique incidence angles. Under normal incidence, the incident wave can be naturally reflected back due to the metallic layer at the bottom, maintaining a reflection amplitude close to unity. Similar to planar plate, the reflection of the metasurface decreases at large angles, but this is one of the important information for obtaining ISAR images corresponding to the structure. It is worth mentioning that there is no supercell in our design, and the phase can be tuned as a linear function of frequency with different slopes. Since the resonance of the meta‐atoms can be precisely adjusted through structural parameters, it is anticipated to be applicable for illusion design in ISAR imaging. By optimizing the meta‐atomic structural parameters, it is possible to achieve the desired phase dispersion over a wide bandwidth and maintain phase insensitivity over a wide angular range. Considering the practical application as well as the phase dispersion characteristics of the meta‐atom, the frequency and angular ranges are set to X‐band (8 to 12 GHz) and −30 to 30°, respectively.^[^ ^51^, ^52^, ^54^ ^]^

The main prerequisite for realizing the metasurface illusion with ISAR imaging is to establish and generalize the relationship between characteristics of meta‐atoms and the position of illusions. Meta‐atoms at different locations of the metasurface can be regarded as different point sources. The total scattered field of the metasurface behaves as a superposition of scattered waves emitted from different characteristic point sources, as shown in Figure 2b. As the incident electromagnetic wave hits the metasurface, the scattered energy radiates in all directions. In this paper, we focus on monostatic ISAR imaging, where the radar transmitter and the receiver are collocated. The far‐field scattered field from the metasurface in the spatial‐frequency *k* domain at an azimuth angle *ϕ* can be expressed as follows:

(1) $$E_{s}\left(k,\phi \right)≅\sum_{i=1}^{n}A_{i}\cdot \exp\left(-j2\vec{k}\cdot {\vec{r}}_{i}+\varphi_{i}\right)$$

where *E_s_* is the total scattering field of the metasurface, *A_i_* is the scattered field amplitude for the *i*‐th meta‐atom of the metasurface, *n* is the number of meta‐atoms, $\vec{k}$ is the vector wave number in the propagation direction, ${\vec{r}}_{i}=x_{i}\;\cdot \hat{x}+y_{i}\cdot \hat{y}$ is the displacement vector from the origin to the location of the *i*‐th scattering center, *φ_i_* is the additional phase regulation introduced by the *i*‐th meta‐atom. In ISAR image, the range is the axis parallel to the direction of radar propagation toward the target, while the cross‐range is the axis perpendicular to the direction of the range. In other words, the range distance *x* is associated with the frequency *f*, and the cross‐range variable *y* is associated with the azimuth angle *ϕ*.

The inverse Fourier transform of the scattered field yields the ISAR image of the structure, and considering the practical case of finite bandwidth B and finite angle Ω, the ISAR image is computed with the scattered field as follows:

(2) $$\begin{array}{ccc} ISAR\left(x,y\right) & = & {F_{k,\phi }}^{-1}\;\left\{E_{s}\left(k,\phi \right)\right\}=\sum_{i=1}^{n}A_{i}\;\\ & & \cdot sinc\left(\frac{2B}{c}\cdot \left(x-x_{i}-\frac{l_{i}c}{4\pi }\right)\right)\cdot sinc\left(\frac{2f_{c}}{c}\Omega \cdot \left(y-y_{i}\right)\right) \end{array}$$

where *l_i_* is the phase dispersion linearity parameter of the *i*‐th meta atom, *f_c_* is the center frequency. The detailed derivation of the process is described in Note S1 (Supporting Information).

It can be seen from Equation (2) that the phase dispersion properties of the meta‐atoms under broadband directly affect the position of the illusions in ISAR image. The displacement distance of the illusion is directly related to the phase dispersion linearity parameter of the meta‐atoms in the broadband, as shown in Figure 2c. A meta‐atom with a phase dispersion linear parameter of *l_i_* results in an illusion with a distance adjustment in ISAR image of Δ*d_i_* = *l_i_c*/4*π*. Detailed calculations and processes for ISAR imaging are described in Note S2 (Supporting Information), with a brief discussion of the factors that determine resolution limits in both the range and cross‐range directions and their implications for illusion fidelity.

**Figure 3:**
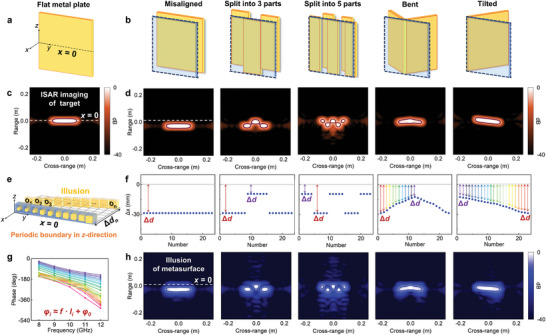
ISAR images of targets with different structural features and illusions. a) Schematic of a flat metal plate placed at *x* = 0. b) Schematic of flat metal plates with different structural features, for instance, metal plates that are misaligned, split into three segments, split into five segments, bent and tilted. c) Calculated ISAR image of a metal plate placed at the *x* = 0. d) Computational ISAR images of metallic flat plates with different structural features. e) Schematic of the metasurface design for the illusion. f) Design of structural positions for different illusions. g) Phase distribution of different meta‐atoms at the broadband. h) ISAR images corresponding to different illusion designs. The dark‐colored lines around structures in the ISAR images are contour lines added by profile processing after imaging to facilitate the observation of the number and features of structures.

A metal plate placed at the *x* = 0 is used as a comparison, which is shown in **Figure** **3**a. Metal plates with different structural features are selected as target objects for the illusion metasurface, as shown in Figure 3b. The computed ISAR image of flat metal plate in Figure 3a is shown in Figure 3c. The simulation and ISAR imaging of the metal plates placed at different locations with different structural features are deployed, as shown in Figure 3d. The ISAR image can visualize the positional information and structural features of various objects, including the misalignment, three‐segment splitting, five‐segment splitting, bending, and tilting of the flat metal plates. In this study, we used “dB” as the unit of ISAR image and normalized it. The effect of the normalization process in mitigating the effects of clutter in ISAR imaging is briefly discussed in Note S3 (Supporting Information).

During the design and computing of various illusions, the total size of the metasurface is constant and always placed at the *x* = 0. The meta‐atoms on the metasurface are distributed periodically along the *z* direction, as shown in Figure 3e. We obtain the positional information of the expected illusions from the structural model, as shown in Figure 3f. Different illusionary effects can be designed by adjusting each meta‐atom holistically, sub‐regionally, and independently. Based on the correlation between the dispersion properties of the meta‐atoms and the displacement distances of the illusions in the ISAR image, we search the structural database for meta‐atoms with desired phase dispersion properties. The theoretical reflectance phase spectra of meta‐atoms used in the design are shown in Figure 3g, which can be expressed as linear functions of frequency. Broadband wide‐angle scattered field data corresponding to each metasurface are collected to compute the corresponding ISAR images. Figure 3h shows metasurface illusions realized with ISAR imaging, demonstrating the misalignment, splitting, bending, and tilting of the metal plate. The metasurfaces achieve illusionary effects with ISAR imaging, which is consistent with designed images in Figure 3d.

Each meta‐atom is designed independently and provides a different localized scattering modulation field. Different dispersion properties of meta‐atoms corresponding to different illusionary effects can be directly observed in imaging. In principle, based on the illusion concept proposed in this paper, the position of the illusions in ISAR image can be adjusted arbitrarily by the design of meta‐atoms. The quantitative assessment used to evaluate the differences between the realized illusions and theoretical predictions can be divided into two main steps. The first step is to verify whether the number of illusions matches the design target. The second step is to extract the intensity distribution of ISAR imaging in the range direction to confirm whether locations of the illusions align with the designed values. The metasurface design allows the adjustment of the exact position of the misaligned illusions observed in ISAR image, as shown in Note S4 (Supporting Information). The design of the illusions with different segmented features is described in Note S5 (Supporting Information). The orientation and angle of the illusions can also be adjusted by metasurface design, as shown in Note S6 (Supporting Information). In addition to the illusions described in this paper, other forms of illusions can be customized with a strong degree of freedom in the design.

### 2.3 Experimental Configurations and Results

**Figure 4:**
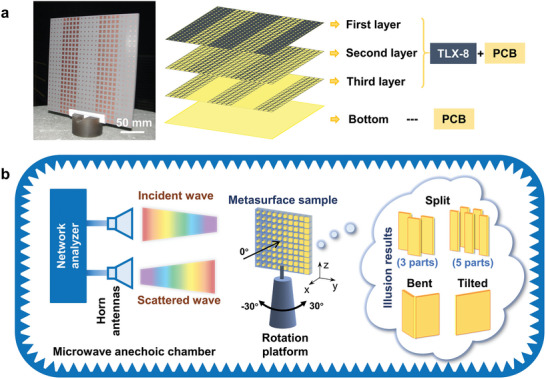
Metasurface sample and schematic of the ISAR imaging experimental setup. a) Diagram of the metasurface sample. The metasurface sample contains three structural layers and a bottom metal layer. Each structural layer contains metal microstructure patches. b) Diagram of the experiment setup of ISAR imaging. The experiment is carried out in a microwave anechoic chamber with two horn antennas for transmitting and receiving broadband electromagnetic waves. A rotation platform controls the rotation of the sample and is used to change the angle of incidence onto the sample. The collected scattered waves are post‐processed to obtain ISAR image using a vector network analyzer.

In the experiment, we fabricate multiple metasurface samples consisting of a three‐layer structure, as shown in **Figure** **4**a. The sample consists of 23 × 23 meta‐atoms with a total area of 184 mm × 184 mm. All microwave samples are fabricated with standard printed circuit board methods. The illusionary performance of the metasurface in ISAR imaging is experimentally validated.

The experimental system of ISAR imaging is carried out in an anechoic chamber to avoid interferences from the environment, as shown in Figure 4b. As the operation bandwidth of the metasurface is X‐band (8–12 GHz), high gain horn antennas working at this frequency range are used as the transmitter and receiver. The transmitting horn antenna and the receiving horn antenna are fixed at the same location and connected to a vector network analyzer to detect the scattered field, including the information of amplitude and phase. In the measurement, we fix the metasurface sample on a rotating platform. The distance between the sample and horn antenna is set to 6 m so that the incident waves can be approximated as plane waves in the far‐field region. To avoid the impact of reflected clutter from the ground, the rotating platform is placed 2.5 m above the ground. Prior to the start of the test, we adjust the placement of the samples. When the transmitting antenna, the receiving antenna, and the central point of the metasurface sample are kept on the same vertical plane, the rotating platform is 0° at this position. During the test, the rotating platform is digitally controlled to rotate within a set test angle range from −30° to 30° with an interval of 0.5°. The image and detailed description of the actual experimental environment and setup are provided in Note S7 (Supporting Information), while the ISAR imaging experiments are performed on different metal target samples in Note S8 (Supporting Information).

**Figure 5:**
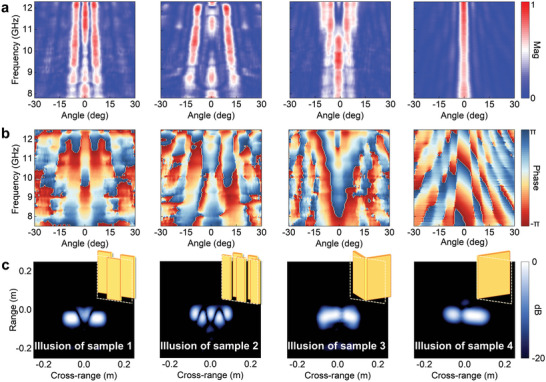
Measured scattered field distribution and ISAR images for the illusions. Distributions of the a) amplitude and b) phase of scattered fields modulated by the metasurface versus the detection frequency and angle. c) ISAR images of different metasurface samples show the illusion of split, bent, and tilted metal plates. A schematic of the reference target in the design is shown in the upper‐right corner of each experimental ISAR image.

The magnitude and phase of scattered fields for different metasurface samples are experimentally scanned over broadband and wide‐angle ranges, whose distributions are shown in **Figure** **5**a,b. The inverse Fourier transform and post‐processing of the scattered fields are performed to obtain ISAR images of matesurface samples. As seen in Figure 5c, the scattered field reconstructed from the first metasurface is imaged as three segmented plates, with the plates at the left and right ends moving downward with respect to the middle plate. The second sample presentation features a five segmented illusion with an axisymmetric distribution in ISAR image, with different positions of structures showing different front‐to‐back travel distances. The third metasurface sample is imaged as a bent flat plate. The fourth metasurface sample is imaged as a tilted flat plate with a designed tilt angle. As seen from those experimental ISAR images, designed metasurfaces show diverse and clear illusionary properties. Dimensional parameters of individual metasurface samples and more experimental results are given in Notes S9 and S10 (Supporting Information), respectively.

## 3 Conclusion

In conclusion, we present a general strategy for realizing metasurface illusion with ISAR imaging. In contrast to previous studies in which illusion discussions have conducted by observing reconstructed electromagnetic field distributions, we define a new illusion paradigm to the direct design of illusions in imaging observations. The implementation of the strategy is based on the construction of a relationship between the meta‐atomic phase dispersion and the displacement distance of the illusion. To explore the capabilities of our approach, we designed metasurfaces enabling the customization of various illusionary effects, such as misaligned metal plates, split metal plates, bent and tilted metal plates. Simulation and experimental results show that the illusion metasurfaces effectively reconstructs the scattered field and presents the designed illusions in ISAR images as if the actual metallic structures are directly observed by a microwave detector. Compared with previous illusion studies at fixed, single frequencies and angles, our research can be applied to broadband and wide‐angle. In addition, our work also witnesses the interplay between the illusion research and radar applications, providing insights and a better understanding of how metasurfaces can contribute to real‐world illusion applications.

The frequency operating range of the illusion metasurface is inherently limited by the resonance structure rather than by the correspondence between the phase features and the illusions in ISAR image. Thus, our strategy also facilitates the design of illusion devices that can be applied to other frequency ranges. The proposed design strategy can be adapted to create illusions for larger objects. For such cases, the number of meta‐atoms would need to be increased to ensure the metasurface fully covers the hidden object. In ISAR imaging, expanding the imaging range does not require an increase in bandwidth or angular range, only an increase in the number of sampling points within a defined frequency and angular range. Additionally, the illusion strategy discussed in this work focuses on modulating the position of the illusion in ISAR imaging rather than eliminating structures and therefore does not involve a discussion of the invisibility case.

We believe that this approach can be further extended to 2D metasurface illusions. In this study, the meta‐atoms on the metasurface are arranged periodically along the *z*‐axis. By designing and adjusting the distribution of meta‐atoms in the *z*‐axis direction and observing the metasurface through 3D ISAR imaging, it would be possible to achieve 2D metasurface illusions. Moreover, polarization is also expected to serve as an additional degree of freedom for the illusion modulation in this strategy. When meta‐atoms are designed to exhibit different phase characteristics under different polarizations, it would be possible to achieve distinct illusionary effects for each polarization through careful design and tuning. This topic is expected to be deeply explored in future studies. We also look forward to more other existing or yet‐to‐be‐conceived properties, such as free‐form illusion meta‐devices, environmentally‐adaptive illusion meta‐devices, and so forth.

## 4 Experimental Section

### Simulation of Broadband Wide‐Angle Scattered Fields with ISAR Imaging

Calculation of the far‐field scattered field of the target over a wide bandwidth and wide‐angle was done by a homemade software named *WGallop*. Considering the symmetry of the target structure, in order to reduce the amount of computation and shorten the computation time, a periodic boundary was used in the direction of the *z*‐axis direction, and an open boundary was used in the direction of the *x*‐axis and *y*‐axis in the computation process. To simulate the equivalent rotational model in ISAR imaging, the frequency scanning range was set to 7.5−12.5 GHz, and the azimuth scanning range was −30°–30°. Inverse Fourier transform and post‐processing of scattered fields from target objects and metasurfaces under broadband and wide‐angle conditions were performed to obtain ISAR images of the target object and realized illusion. ISAR images of the 1D metasurface illusion were generated using the built‐in ISAR imaging module of *WGallop*. The simulation time varies depending on the simulation setup, the desired accuracy, the number of meshes, and the available hardware configuration. For the setup, the study used a 3.4‐GHz Intel Xeon E3‐1231 V3 processor with 16 GB of RAM, and the simulations required a couple of hours to over a dozen hours. When considering the 2D metasurface illusion, the meta‐atoms were no longer periodically distributed in the *z*‐axis, at which point periodic boundary conditions cannot be used and the number of meshes in the structure increases. In addition, on the basis of 2D ISAR imaging, 3D ISAR imaging requires the calculation of the scattered field distribution at different pitch angles. As a result, the computation time for 2D metasurface illusion will increase accordingly.

### Scattered Field Collection and ISAR Imaging of Experimental Metasurface Samples

A broadband amplitude‐phase measurement system using a vector network analyzer as the core was used. During the imaging test, the metasurface sample was placed on a rotating platform and rotated according to the set azimuth angle. The vector network analyzer synchronously acquires the amplitude and phase signals of the echo sweep of the sample under test at set angular intervals until the entire azimuthal range is covered. The acquired echo data was 2D, with one dimension varying with azimuth and the other with frequency. Through data calibration, background filtering, and imaging processing, a 2D ISAR image of the sample can be acquired.

## Conflict of Interest

The authors declare no conflict of interest.

## Author Contributions

H.L. and K.L. contributed equally to this work. H.L., Y.S., and Z.W. conceived the idea. H.L., Y.L., and Z.W. performed numerical simulations. H.L., L.D., Y.F., and Z.W. conducted the experiment. All authors were involved in the discussion and analysis. H.L., K.L., D.P.T., Y.L., L.D., S.L., M.K.C., Y.S., Z.W., and X.C. prepared the manuscript. D.P.T., Y.S., Z.W., and X.C. coordinated all the work.

## Supporting information



Supporting Information

## Data Availability

The data that support the findings of this study are available from the corresponding author upon reasonable request.
