# Supplementary material for: Broadband, Wide‐Angle, and Versatile Metasurface Illusions with Inverse Synthetic Aperture Radar Imaging
Source: Adv Sci (Weinh). 2025 Feb 8;12(18):2416172. doi: 10.1002/advs.202416172 (PMC12079480; doi:10.1002/advs.202416172)
Supplement: Supplementary file 1 — Supporting Information [file ADVS-12-2416172-s001.pdf]

ADVANCED SCIENCE
Open Access

Supporting Information

for *Adv. Sci.*, DOI 10.1002/advs.202416172

Broadband, Wide-Angle, and Versatile Metasurface Illusions with Inverse Synthetic Aperture Radar Imaging

Haoran Li, Kang Luo, Din Ping Tsai, Yulang Li, Linyuan Dou, Shuqiao Li, Tao He, Yuancheng Fan, Mu Ku Chen, Zhanshan Wang, Yuzhi Shi*, Zeyong Wei* and Xinbin Cheng**

Supporting Information

Broadband, Wide-angle and Versatile Metasurface Illusions with Inverse Synthetic Aperture Radar Imaging

Haoran Li, Kang Luo, Din Ping Tsai, Yulang Li, Linyuan Dou, Shuqiao Li, Tao He, Yuancheng Fan, Mu Ku Chen, Zhanshan Wang, Yuzhi Shi*, Zeyong Wei* and Xinbin Cheng**

Supplementary Note 1: Derivation of correspondence between the dispersion properties of metasurface and the displacement of illusion in ISAR imaging process

Inverse synthetic aperture radar (ISAR) is a widely used radar imaging technique. Extensive researches have been conducted to discuss it and its related applications.[1-4] An ISAR image can be viewed as range and cross-range profiles of a target displayed on a two-dimensional (2D) plane. The range profiles are obtained by processing the scattered fields form a single angle with different frequencies, whereas the cross-range profiles are obtained by processing the scattered from one frequency with different angles.

There exist n meta-atoms on the metasurface, each of which can be regarded as a point scatterer at different location. The scattered electric field in the far field can be approximated as the summation of each point scatters,

$$E_s(k,\phi) \cong \sum_{i=1}^{n} A_i \cdot \exp\left(-j2\vec{k}\cdot\vec{r}_i + \varphi_i\right), \tag{S1}$$

where $k = 2\pi f/c$ is the wave number for the frequency f, ϕ is the angle. A_i is the amplitude of the scattered electric field for the i-th meta-atom, $\vec{k}$ is the vector wave number in the propagation direction, $\vec{r}_i$ is the displacement vector from the origin to the location of the i-th meta-atom, φ_i is the additional phase regulation introduced by the i-th meta-atom. The phase reference plane of the incident wave is in the $x = 0$ plane.

In the 2D space, $\vec{k}\cdot\vec{r}_i$ can be written as

$$\begin{aligned}\vec{k}\cdot\vec{r}_i &= \left(k_x\hat{x} + k_y\hat{y}\right)\cdot\left(x_i\hat{x} + y_i\hat{y}\right)\\ &= k_x \cdot x_i + k_y \cdot y_i\\ &= k\cos\phi\cdot x_i + k\sin\phi\cdot y_i,\end{aligned} \tag{S2}$$

where $\hat{k}$, $\hat{x}$, and $\hat{y}$ are the unit vectors in k, x, and y directions, respectively. Therefore, Equation S1 can be written as

$$\begin{aligned} E_s(k,\phi) &\cong \sum_{i=1}^{n} A_i \cdot \exp\left(-j2\vec{k}\cdot\vec{r_i}+\varphi_i\right) \\ &= \sum_{i=1}^{n} A_i \cdot \exp\left(-j2\left(k\cos\phi\cdot x_i + k\sin\phi\cdot y_i\right)+\varphi_i\right) \\ &= \sum_{i=1}^{n} A_i \cdot \exp\left(-j2k\cos\phi\cdot x_i+\varphi_i\right)\cdot\exp\left(-j2k\sin\phi\cdot y_i\right), \end{aligned} \tag{S3}$$

where the meta-atomic reflection phase characteristic denoted by φ_i acts only in the x-direction. For small values of ϕ, cos ϕ and sin ϕ are approximated to 1 and ϕ, respectively. Therefore, Equation S3 can be written as

$$\begin{aligned} E_s(k,\phi) &= \sum_{i=1}^{n} A_i \cdot \exp\left(-j2\pi\left(\frac{2f}{c}\right)\cdot x_i+\varphi_i\right)\cdot\exp\left(-j2\pi\left(\frac{2f}{c}\right)\phi\cdot y_i\right) \\ &= \sum_{i=1}^{n} A_i \cdot \exp\left(-j2\pi\alpha\cdot x_i+\varphi_i\right)\cdot\exp\left(-j2\pi\gamma\cdot y_i\right), \end{aligned} \tag{S4}$$

where $2f/c \triangleq \alpha$ and $2f/c\cdot\phi \triangleq \gamma$. The range and cross-range profile can be constructed by taking the iterative Fourier transform (IFT) with respect to α and γ. The ISAR image in x-y plane can be obtained by taking the 2D IFT as

$$\begin{aligned} E_s(x,y) &= \mathcal{F}_{\alpha,\gamma}^{-1}\left\{E_s(k,\phi)\right\} \\ &= \sum_{i=1}^{n} A_i \cdot \mathcal{F}_{\alpha}^{-1}\left\{\exp\left(-j2\pi\alpha\cdot x_i+\varphi_i\right)\right\}\cdot\mathcal{F}_{\gamma}^{-1}\left\{\exp\left(-j2\pi\gamma\cdot y_i\right)\right\} \\ &\triangleq ISAR(x,y), \end{aligned} \tag{S5}$$

where $\mathcal{F}_{\alpha,\gamma}^{-1}$ denotes the IFT operation with respect to α and γ. For ease of calculation and observation, we separate the integration of frequency and angle. We collected the scattered field data within a finite bandwidth B of frequencies ranging from $f_{\min}$ to $f_{\max}$. ISAR(x) represents the range profile as a function of range x.

$$\begin{aligned} ISAR(x) &= \mathcal{F}_{\alpha}^{-1}\left\{\sum_{i=1}^{n}\exp\left(-j2\pi\alpha\cdot x_i+\varphi_i\right)\right\} \\ &= \sum_{i=1}^{n}\int_{\alpha_{\min}}^{\alpha_{\max}}\left[\exp\left(-j\left(2\pi\alpha\cdot x_i+\varphi_i\right)\right)\right]\exp\left(j2\pi\alpha\cdot x\right)d\alpha \\ &= \sum_{i=1}^{n}\int_{\alpha_{\min}}^{\alpha_{\max}}\left[\exp\left(-j\left(2\pi\alpha\cdot x_i+f\cdot l_i+\varphi_0\right)\right)\right]\exp\left(j2\pi\alpha\cdot x\right)d\alpha, \end{aligned} \tag{S6}$$

where, $\alpha_{\min} = 2f_{\min}/c$, $\alpha_{\max} = 2f_{\max}/c$, $\varphi_i \approx f\cdot l_i + \varphi_0$, l_i represents the linear parameter of the phase that transforms linearly with frequency f and φ_0 represents the portion of the phase that is independent of frequency change which has no effect on the results of the inverse Fourier variation. We present φ_0 outside the integral and perform a definite integral over the ISAR(x),

Equation S6 can be written as

$$\begin{aligned} ISAR(x) &= \sum_{i=1}^{n} \exp\left(-j\varphi_0\right)\cdot\int_{\alpha_{\min}}^{\alpha_{\max}} \exp\left(j2\pi\alpha\left(x - x_i - \frac{l_i c}{4\pi}\right)\right)d\alpha \\ &= \left(\frac{2B}{c}\right)\sum_{i=1}^{n}\exp\left(j2k_c\left(x - x_i - d_i\right) - j\varphi_0\right)\cdot \sin c\left(\left(\frac{2B}{c}\right)\cdot\left(x - x_i - d_i\right)\right), \end{aligned} \tag{S7}$$

where $d_i = l_i c/4\pi$ denotes the moving distance of the illusion in ISAR image due to the phase dispersion of the i-th meta-atom. $k_c = 2\pi f_c/c$ is the wave number corresponding to the center frequency $f_c = (f_{\min} + f_{\max})/2$. Here, sinc function is the amplitude term that specifies the shape function of the meta-atom.

We collected the scattered field data within a finite angular range Ω from –Ω/2 to Ω/2. ISAR(y) represents the cross-range profile function as a function of y. Since the meta-atom does not provide additional dispersion modulation in the y direction, the definite integral can be calculated as

$$\begin{aligned} ISAR(y) &= \mathcal{F}_{\gamma}^{-1}\left\{\sum_{i=1}^{n}\exp\left(-j2\pi\gamma\cdot y_i\right)\right\} \\ &= \sum_{i=1}^{n}\int_{-\gamma_m/2}^{\gamma_m/2}\left[\exp\left(-j2\pi\gamma\cdot y_i\right)\right]\exp\left(j2\pi\gamma\cdot y\right)d\gamma \\ &= \sum_{i=1}^{n}\int_{-\gamma_m/2}^{\gamma_m/2}\left[\exp\left(j2\pi\gamma\cdot\left(y - y_i\right)\right)\right]d\gamma \\ &= \left(\frac{2f_c}{c}\Omega\right)\cdot\sum_{i=1}^{n}\sin c\left[\frac{2f_c}{c}\Omega\left(y - y_i\right)\right], \end{aligned} \tag{S8}$$

where $\gamma_m = 2f\Omega/c$. Then, the ISAR image of the metasurface can be calculated by taking the 2D inverse Fourier integral of the scattered field as

$$\begin{aligned} ISAR(x,y) &= \mathcal{F}_{\alpha,\gamma}^{-1}\left\{E_s(k,\phi)\right\} \\ &= \sum_{i=1}^{n} A_i\cdot\sin c\left(\frac{2B}{c}\cdot\left(x - x_i - d_i\right)\right)\cdot\sin c\left(\frac{2f_c}{c}\Omega\cdot\left(y - y_i\right)\right). \end{aligned} \tag{S9}$$

Therefore, the scattering of meta-atoms centered at the illusion locations of "$x_i + d_i$" on the range and true locations of "y_i" on the cross-range. In this way, the x_i locations of the true structures cannot be resolved. The sinc defocusing around the scattering centers is unavoidable due to finite bandwidth of the radar signal.

The approximate first-order linear relationship does not hold at broadband and wide-angle, and direct 2D inverse Fourier variation of the fan-distributed data results in migrations of scattering points away from the image center, leading to image distortion. To solve this problem, the data were interpolated and the echo data were acquired into a rectangular grid to enable the desired ISAR image.

Supplementary Note 2: Computational flow of ISAR imaging process and parameter settings

The process of ISAR imaging is relatively standardized[4] and the individual steps of the algorithm for performing ISAR imaging calculations on object are briefly described in order, as shown in **Figure S1**.

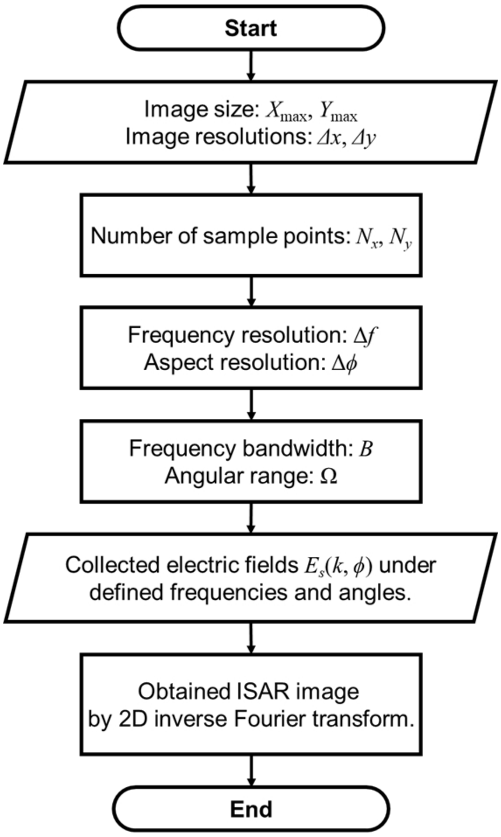

Figure S1. Flowchart of ISAR imaging calculations.

The size of the ISAR image (the range and cross-range window extends) is selected based on the object size. In this study, the size of the ISAR image (X_{max} by Y_{max}) is chosen to be 0.5 m × 0.5 m to cover the actual size of the object to be imaged.

The range resolutions Δx and cross-range resolutions Δy define the number of pixels used for imaging. Once the resolution of the ISAR image is determined, the number of sample points N_x required within the test range and the number of sample points N_y within the cross-range can be calculated:

$$N_x = \frac{X_{max}}{\Delta x}, \tag{S10}$$

$$N_y = \frac{Y_{max}}{\Delta y}. \tag{S11}$$

The frequency resolution Δf and the angle resolution $\Delta\phi$ are determined based on the

Fourier relationship as

$$\Delta f = \frac{B}{N_x} = \frac{c/2}{X_{\max}}, \tag{S12}$$

$$\Delta \phi = \frac{\Omega}{N_y} = \frac{\lambda_c/2}{Y_{\max}}, \tag{S13}$$

where λ_c represents the wavelength corresponding to the center frequency. The relationship between the frequency bendwidth B and angular range Ω with the parameters of ISAR image can be described as follows:

$$B = N_x \cdot \Delta f = \frac{N_x \cdot c}{2 \cdot X_{\max}}, \tag{S14}$$

$$\Omega = N_y \cdot \Delta \phi = \frac{N_y \cdot \lambda_c}{2 \cdot Y_{\max}}. \tag{S15}$$

Based on the relationship above, it can be found that when the frequency bandwidth is set to B, the range resolution Δx can be calculated with:

$$\Delta x = \frac{X_{\max}}{N_x} = \frac{c/2}{\Delta f} \cdot \frac{\Delta f}{B} = \frac{c}{2B}. \tag{S16}$$

Our study is conducted in the X-band, the frequency range is set to 8–12 GHz and the center frequency is 10 GHz. Thus, we can determine that the resolution Δx of the image in the corresponding range is 0.0375m.

The relationship between the cross-range resolution Δy and angular range Ω can also be summarized as

$$\Delta y = \frac{Y_{\max}}{N_y} = \frac{\lambda_c/2}{\Delta \phi} \cdot \frac{\Delta \phi}{\Omega} = \frac{\lambda_c}{2\Omega}. \tag{S17}$$

The angular range from −30° to 30° and the center angle is 0° in our study, and the corresponding ISAR image has an image resolution Δy about 0.015 m over the angular range.

As the ISAR image resolutions are determined, the number of sample points N_x required within the test range and the number of sample points N_y within the cross-range are also determined. The frequency and angle required for ISAR imaging process can then be specified correspondingly. During the setup, the radar transmits wave signals at N_x different frequencies ($f_{\min}$,$f_{\min} + \Delta f$, ...,$f_{\max}$) and N_y different angles (–Ω/2, –Ω/2 + $\Delta\phi$, ..., Ω/2). The scattered electric field is collected at desired frequencies and angles. Finally, the ISAR image can be obtained through 2D IFT.

The upper limits of the range and cross-range resolution are determined by the modulation capacity of the metasurface and the size of the illusion structure, respectively. Meanwhile, the

lower limit of the resolution factor is determined by the frequency and angular range considered in the study.

The modulation capability of the metasurface influences the displacement of the illusion structure in the range direction of ISAR image. When the resolution in the range direction exceeds the maximum displacement, the modulation of the illusion structure cannot be recognized. In studies considering the realization of multiple illusions, when the cross-range resolution is larger than the size of the illusion structure, illusionary effects of multiple structures cannot be observed.

In ISAR imaging, resolutions in the range and cross-range direction are directly determined by the frequency bandwidth and angular range. A complete ISAR image cannot be obtained when the frequency bandwidth and angular range required for higher resolution exceed practical application and testing capabilities. Furthermore, in the case of larger frequency and angular ranges, there needs to be a discussion about whether the meta-atomic properties can be maintained; otherwise, the illusion design may fail.

Supplementary Note 3: Normalization of ISAR images

Figure S2a presents the raw ISAR imaging results and Figure S2b illustrates the results after normalizing the ISAR image data. The comparison demonstrates that the normalization process effectively eliminates clutter surrounding the structure and at the edges of the image. Imaging of the structure is better demonstrated, while structural features are almost unaffected.

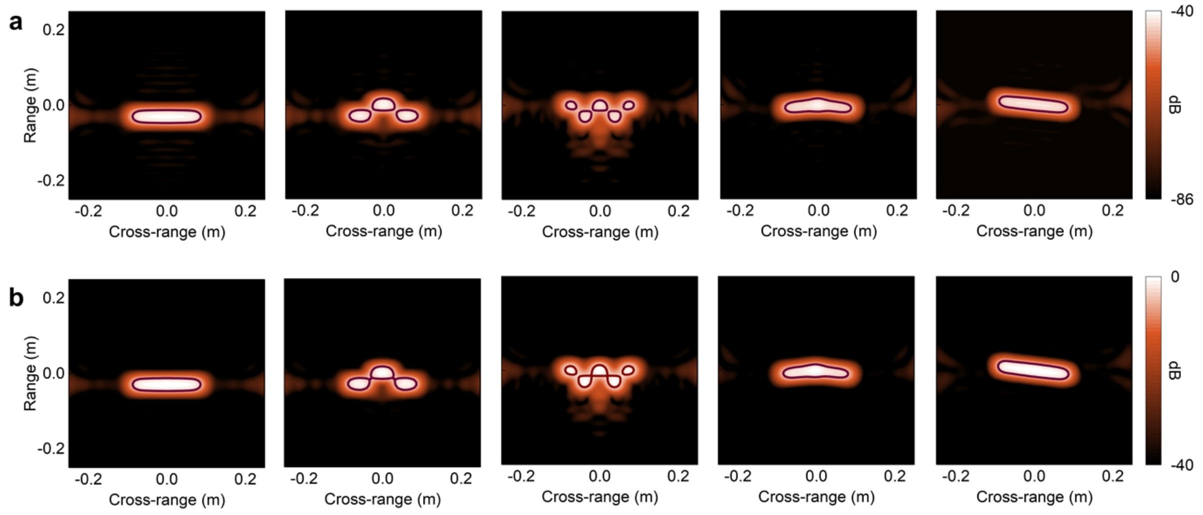

Figure S2. Comparison between the original ISAR image and the normalized processed ISAR image. (a) ISAR images based on raw data. (b) Normalized ISAR image.

Supplementary Note 4: Illusions with freely adjustable movement distance

As shown in **Figure S3**a, when the position of the metal plate is deviated from the $x = 0$, the movement of the structure can be clearly viewed by ISAR images. For the illusion of the misaligned metallic flat plate, the dimensional parameters of the meta-atoms at each location on the metasurface are kept consistent in order to realize the overall displacement of the illusion in the ISAR image. According to the relationship between the linear parameter of the broadband phase variation and the displacement distance, illusions with different movements can be obtained by adjusting the phase dispersion characteristics of the meta-atoms. The multi-layer meta-atoms used in this study enables the illusion to have a distance shift from 7 to 30 mm relative to the metasurface placement position in the ISAR image. Figure S3b shows the misalignment illusions in ISAR images achieved by different metasurface design for translation distances of 10 mm, 15 mm, 20 mm and 25 mm. The accuracy of the illusion location can be clarified by comparing it with ISAR images of metal structures at corresponding placement location, as shown in Figure S3c.

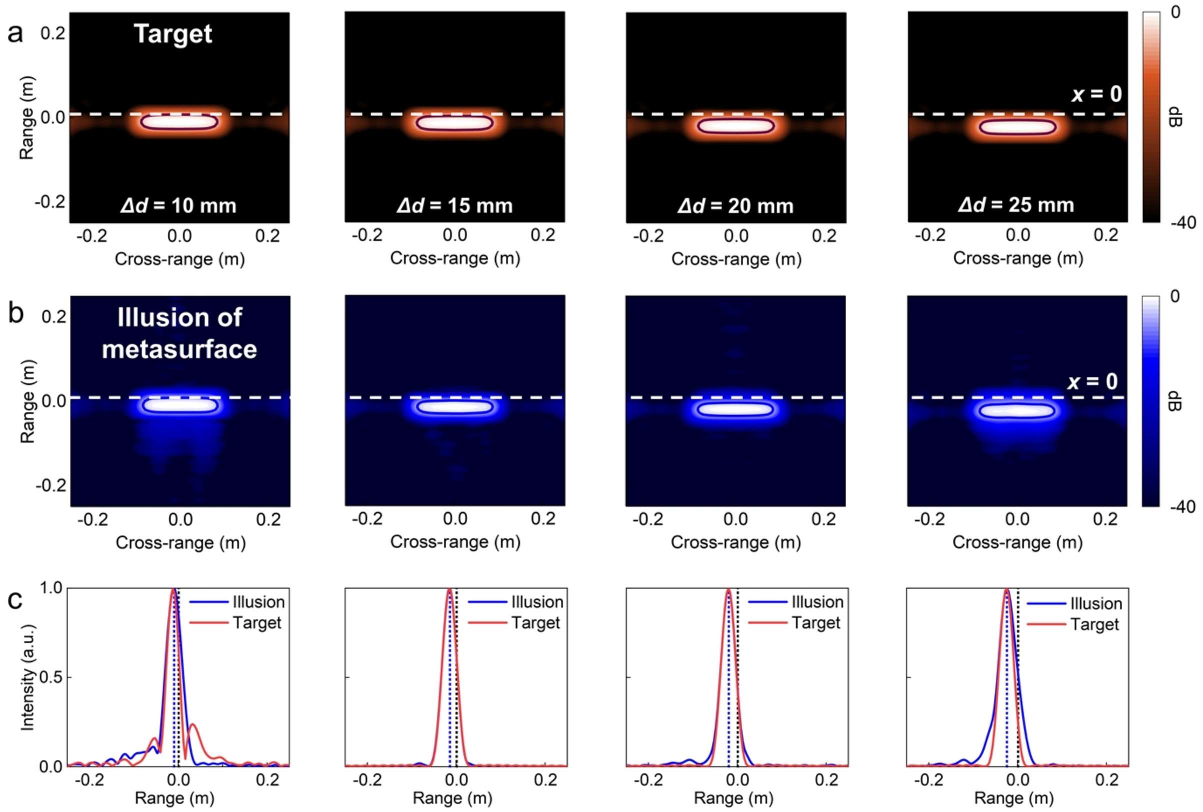

Figure S3. Misaligned illusions with different movement distances. (a) ISAR images of misaligned metal plates with moving distances of 10 mm, 15 mm, 20 mm, and 25 mm with respect to $x = 0$. (b) The designed metasurfaces realize misaligned metal plate illusions with moving distances of 10 mm, 15 mm, 20 mm, and 25 mm with respect to $x = 0$ in ISAR images. (c) Comparison of the position of the misaligned metal plate illusions with the targets.

Supplementary Note 5: Freely customizable segmented illusions

ISAR images of metal plates with different number and positional features are shown in **Figure S4**a. By designing the meta-atoms in different regions individually and keeping the properties of the meta-atoms in the same region consistent, the illusions in ISAR images behave as metallic structures with different positional properties, as shown in Figure S4b.

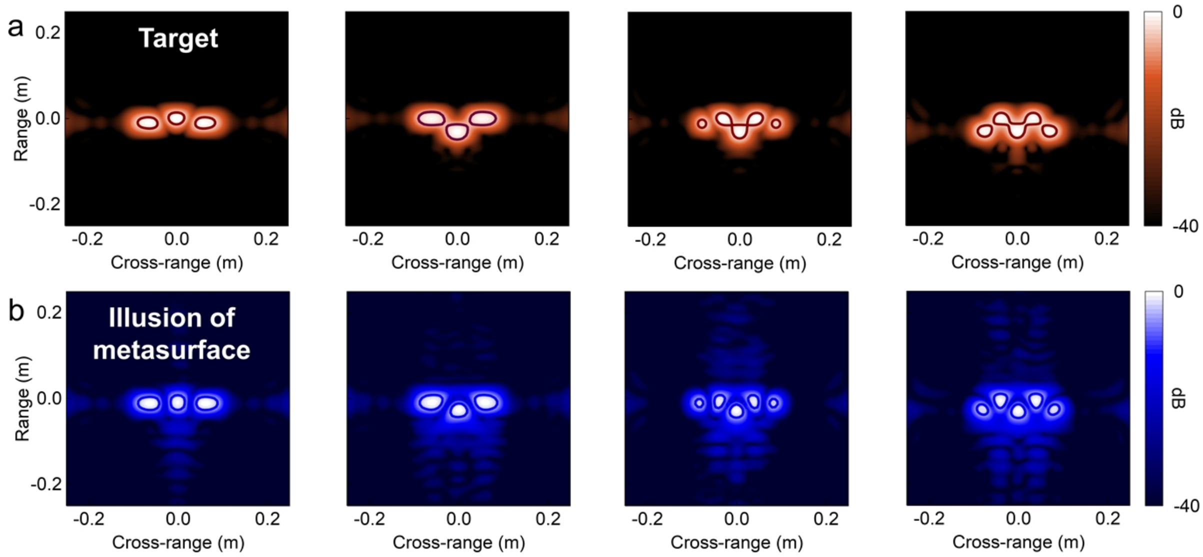

Figure S4. Illusions of custom segmentation design. (a) ISAR images of metal plates with different numbers and positional characteristics. (b) Different segmented metal plate illusions realized by designing metasurfaces with reference to the targets.

Supplementary Note 6: Tilting and bending illusions with adjustable angle and orientation enabled by metasurfaces

The ISAR images of **Figure S5**a and S5b show the structural properties of strongly bent and slightly bent metal plates, respectively. The ISAR images of Figure S5c and S5d show the structural properties of the metal plates with tilt angle of 7° and 2°, respectively. In order to realize the illusion of bent and tilted metal flat plates, we arrange sub-wavelength multi-resonance meta-atoms with different size parameters at different locations. When the slope of the phase spectrum of meta-atoms varies uniformly and symmetrically with the arrangement of the unit strucutres, the illusion of the bent plate can be realized in ISAR image, as shown in Figure S5e. The bend angle of the illusion can be precisely adjusted by changing the gradient of the broadband phase linearity values between different meta-atoms, as shown in Figure S5f. Based on the same design method, by adjusting the arrangement of the meta-atoms, we can design the gradient distribution of the translation distances of the different small illusions. As shown in Figure S5g, the overall illusion in the ISAR image appears as a tilted metal plate. The

tilt angle of the illusion can also be adjusted, as shown Figure S5h. In addition, the tilting direction and bending orientation of the illusion can also be changed by adjusting the arrangement order of different meta-atoms.

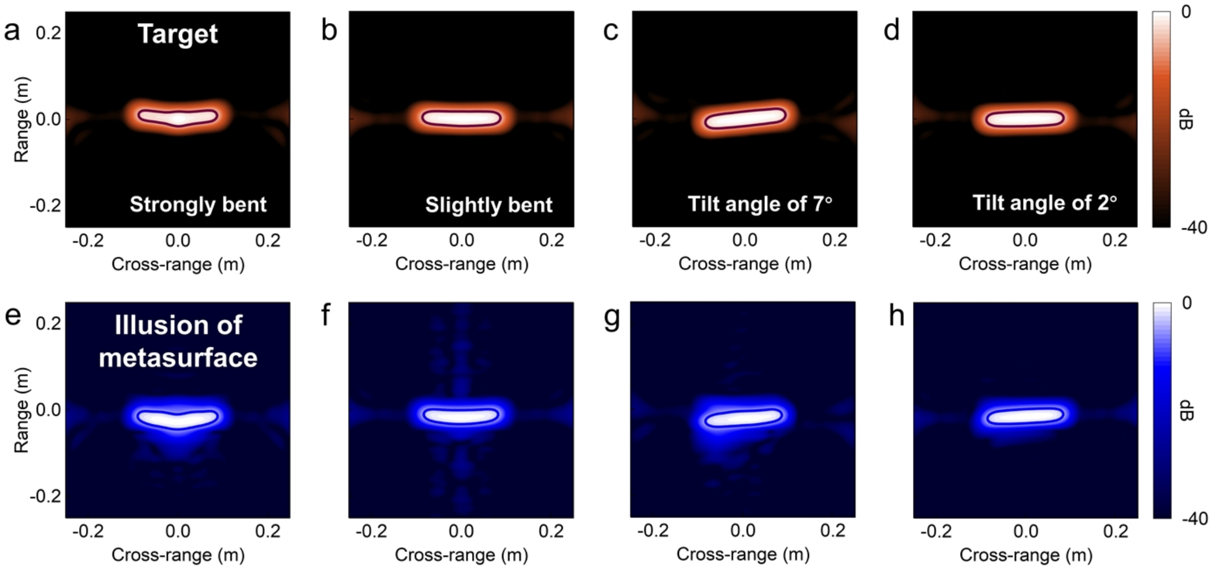

Figure S5. Illusions of tilted and bent flat plates with adjustable orientation and angle by metasurface design. (a), (b) ISAR images of bent metal plates with strong and slight bending properties, respectively. (c), (d) ISAR images of tilted plates with tilt angle of 7° and 2°, respectively. (e), (f) ISAR images of illusions with strong and slight bending properties enabled by the designed metasurfaces, respectively. (g), (h) ISAR images of illusions with tilt angle of 7° and 2° enabled by the designed metasurfaces, respectively.

Supplementary Note 7: Experiment setup of ISAR imaging

The ISAR imaging experimental system is arranged in a microwave anechoic chamber, as shown in **Figure S6**. Tests are performed using a broadband amplitude-phase measurement system centered on a vector network analyzer. 8–12 GHz transmitter horn antenna and receiver horn antenna are fixed in the same location connected to the vector network analyzer. The samples are placed and fixed on a rotation platform at a distance of 6 m from the horn antennas. To avoid the influence of ground reflection clutter, the height of the rotation platform is 2.5 m. During the test, the target on the rotation platform rotates according to the set azimuth angle. The vector network analyzer synchronously collects the amplitude and phase information of the target scattered field at different frequencies according to the set angular interval.

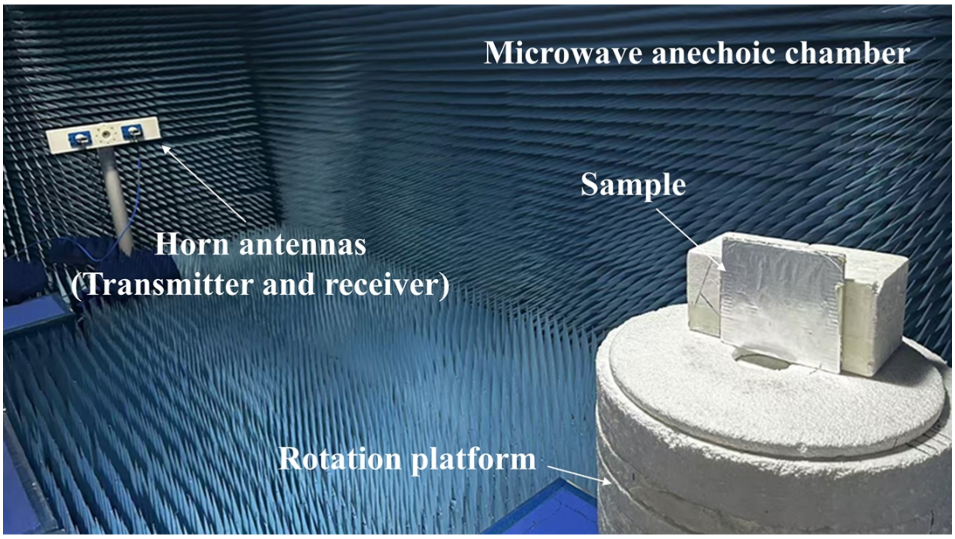

Figure S6. Schematic of the ISAR imaging experimental environment and setup

Supplementary Note 8: ISAR imaging of metal target samples

We employ the experimental procedure to perform ISAR imaging of metallic structures referenced in the illusion design. As shown in **Figure S7**, ISAR images obtained from the experiments clearly reveal the structural features corresponding to the different targets.

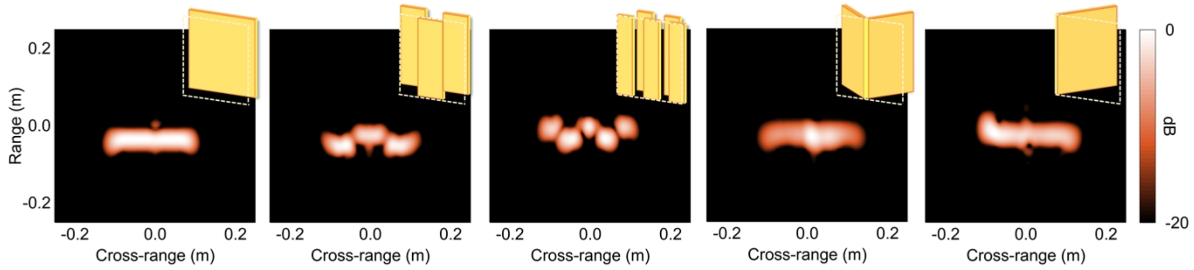

Figure S7. Experimental ISAR images of metallic targets.

Supplementary Note 9: Structural parameters of experimentally metasurface samples

Table S1. Structural parameters of metasurface sample 1.

Number	1	2	3	4	5	6	7	8	9	10	11	12
a_1 (mm)	5	5	5	5	5	5	5	5	2	2	2	2
a_2 (mm)	5	5	5	5	5	5	5	5	6	6	6	6
a_3 (mm)	7.8	7.8	7.8	7.8	7.8	7.8	7.8	7.8	6	6	6	6
Number	13	14	15	16	17	18	19	20	21	22	23	
a_1 (mm)	2	2	2	5	5	5	5	5	5	5	5	
a_2 (mm)	6	6	6	5	5	5	5	5	5	5	5	
a_3 (mm)	6	6	6	7.8	7.8	7.8	7.8	7.8	7.8	7.8	7.8	

Table S2. Structural parameters of metasurface sample 2.

Number	1	2	3	4	5	6	7	8	9	10	11	12
a_1 (mm)	2	2	2	2	5	5	5	5	5	2	2	2
a_2 (mm)	6	6	6	6	5	5	5	5	5	6	6	6
a_3 (mm)	6	6	6	6	7.8	7.8	7.8	7.8	7.8	6	6	6
Number	13	14	15	16	17	18	19	20	21	22	23	
a_1 (mm)	2	2	5	5	5	5	5	2	2	2	2	
a_2 (mm)	6	6	5	5	5	5	5	6	6	6	6	
a_3 (mm)	6	6	7.8	7.8	7.8	7.8	7.8	6	6	6	6	

Table S3. Structural parameters of metasurface sample 3.

Number	1	2	3	4	5	6	7	8	9	10	11	12
a_1 (mm)	5	5	5	4	4	4	4	4	4	4	4	4
a_2 (mm)	5	5	5	5	5	5	4	4	4	4	4	4
a_3 (mm)	7	6.7	6.5	7	6.7	6.5	6.4	6.1	5.8	5.4	4.9	4
Number	13	14	15	16	17	18	19	20	21	22	23	
a_1 (mm)	4	4	4	4	4	4	4	4	5	5	5	
a_2 (mm)	4	4	4	4	4	5	5	5	5	5	5	
a_3 (mm)	4.9	5.4	5.8	6.1	6.4	6.5	6.7	7	6.5	6.7	7	

Table S4. Structural parameters of metasurface sample 4.

Number	1	2	3	4	5	6	7	8	9	10	11	12
a_1 (mm)	1	1	2	1	2	3	3	3	3	4	3	4
a_2 (mm)	1	2	2	3	3	3	3	3	4	4	4	4
a_3 (mm)	1	2.5	3.1	3	3.6	3.7	4.2	4.6	4	4.7	4.9	5.1
Number	13	14	15	16	17	18	19	20	21	22	23	
a_1 (mm)	4	5	5	4	3	3	1	1	1	1	1	
a_2 (mm)	4	5	5	5	5	5	4	4	4	4	4	
a_3 (mm)	5.4	5	5.5	5.8	6.1	6.3	6.5	6.7	6.9	7.1	7.2	

Supplementary Note 10: Complementing multiple illusion metasurfaces design and ISAR imaging experiments

During the experiment, in addition to the four metasurface samples shown in the manuscript. We performed the preparation of additional metasurface samples with ISAR imaging tests. **Figure S8**, **Figure S9** and **Figure S10** show the design, structural parameters, distribution of scattered fields, and ISAR imaging results of the corresponding metasurfaces in the supplemental experiments, respectively.

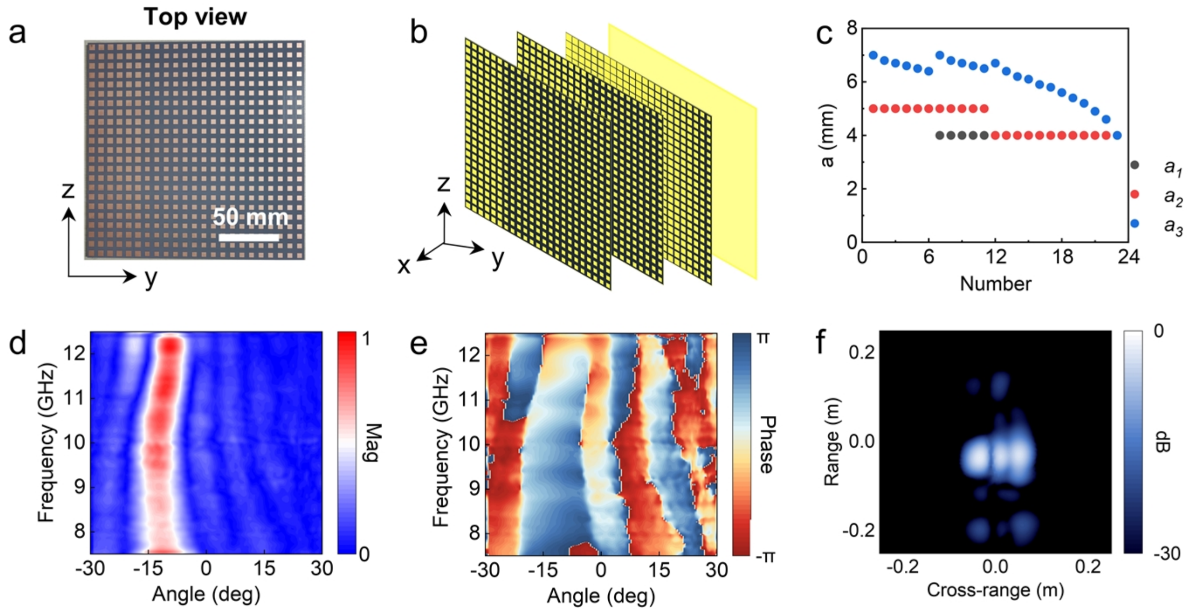

Figure S8. Metasurface design and realization of the tilted illusion. (a) Top view of the metasurface sample. (b) Morphology of the different layers of the sample. (c) Structural parameters of the sample. (d), (e) Magnitude and phase distribution of the scattering field of the metasurface, respectivly. (f) The ISAR image of the metasurface is presented as a tilted plate illusion.

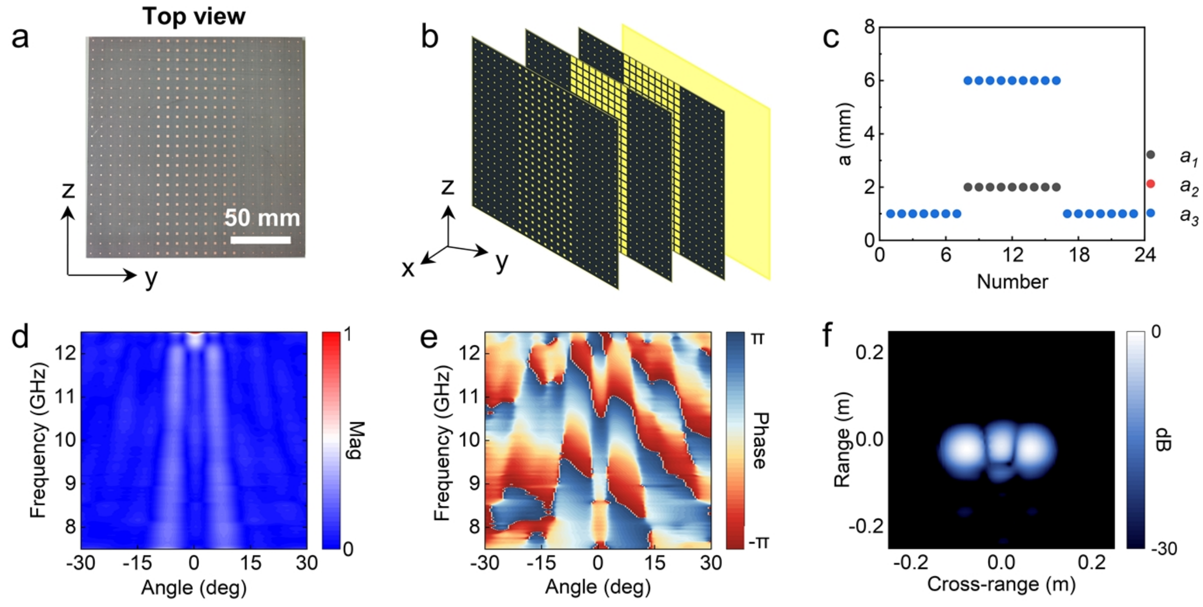

Figure S9. Metasurface design and realization of the illusion with three–segmented property. (a) Top view of the metasurface sample. (b) Morphology of the different layers of the sample. (c) Structural parameters of the sample. (d), (e) Magnitude and phase distribution of the

scattering field of the metasurface, respectivly. (f) The ISAR image of the metasurface is presented as a three-segmented metal plate illusion.

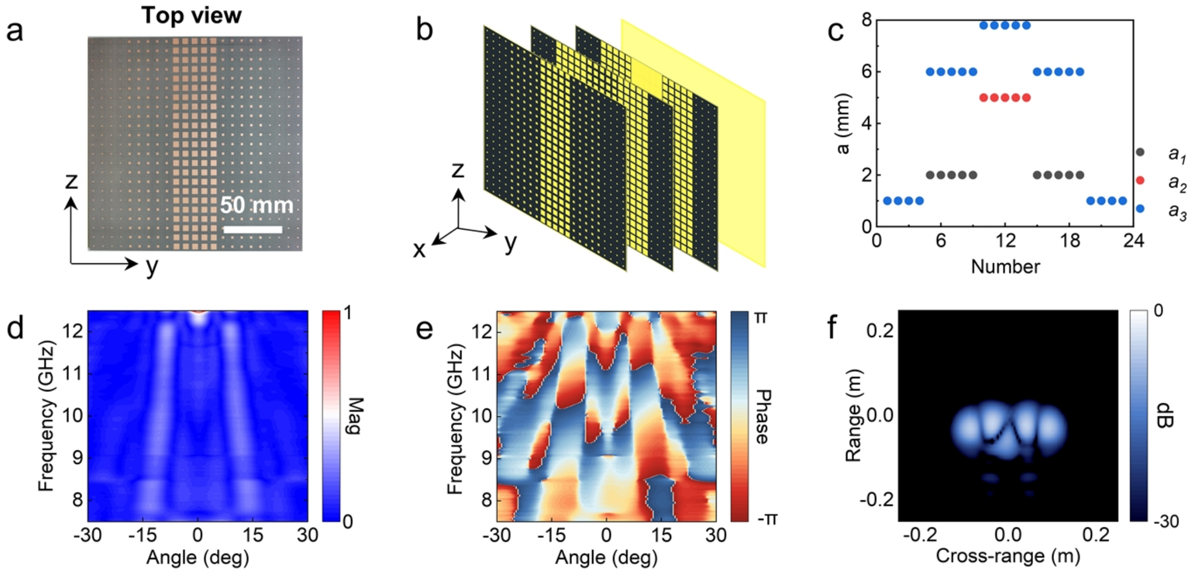

Figure S10. Metasurface design and realization of the illusion with five–segmented property. (a) Top view of the metasurface sample. (b) Morphology of the different layers of the sample. (c) Structural parameters of the sample. (d), (e) Magnitude and phase distribution of the scattering field of the metasurface, respectivly. (f) The ISAR image of the metasurface is presented as a five-segmented metal plate illusion.

References

[S1] D. L. Mensa, *High resolution radar cross-section imaging*, Artech House, **1991**.

[S2] V. Chen, M. Martorella, *Inverse Synthetic Aperture Radar Imaging: Principles, Algorithms and Applications*, IET, **2014**.

[S3] B. R. Mahafza, *Introduction to Radar Analysis (2nd ed.),* Chapman and Hall/CRC, **2017**.

[S4] C. Ozdemir, *Inverse synthetic aperture radar imaging with MATLAB algorithms*, Wiley, **2021**.
